# Traditional Chinese Medicine-Loaded Hydrogels: An Emerging Strategy for the Treatment of Bone Infections

**DOI:** 10.3390/pharmaceutics17040514

**Published:** 2025-04-14

**Authors:** Xueyi Jin, Yujie Yue, Huaanzi Hu, Songwei Lv

**Affiliations:** School of Pharmacy, Changzhou University, Changzhou 213164, China; s24091055015@smail.cczu.edu.cn (X.J.); s23091055043@smail.cczu.edu.cn (Y.Y.)

**Keywords:** Traditional Chinese Medicine (TCM), hydrogel, bone infection, bone regeneration, drug delivery

## Abstract

Bone infection is a disease that seriously affects patients’ quality of life and physical health. Traditional treatment methods have many drawbacks. Hydrogels loaded with Traditional Chinese Medicine (TCM), as an emerging treatment strategy, combine the advantages of good biocompatibility of hydrogels, adjustable drug release performance, and multi-target synergistic treatment of TCM, showing great application potential. This article elaborates in detail on the research progress of hydrogels loaded with TCM for the treatment of bone infections, including the classification and characteristics of hydrogels, the mechanism of action of TCM in the treatment of bone infections, the preparation methods of hydrogels loaded with TCM, application examples, advantages, and the challenges and prospects faced. The aim is to provide new ideas and references for the clinical treatment of bone infections.

## 1. Introduction

Bone infection, or osteomyelitis, is an inflammatory disorder stemming from the invasion of pathogens, including bacteria and fungi, into the bones. Its etiology is multifaceted, commonly associated with trauma, surgical site infections, and hematogenous dissemination. Bone infection can induce local manifestations such as pain, swelling, and fever. In severe instances, it may give rise to bone necrosis, pathological fractures, and potentially life-threatening conditions [1]. Epidemiological data indicate that the global incidence of bone infection has been increasing annually. This trend places a substantial physical, psychological, and economic burden on patients [2]. Conventional treatment modalities for bone infections principally encompass systemic antibiotic administration and surgical intervention [3]. Despite the fact that systemic antibiotic administration can, to a certain degree, inhibit bacterial growth, it is prone to inducing drug resistance and eliciting substantial systemic adverse effects. These effects include impairment of liver and kidney functions as well as disruption of intestinal flora [4]. Surgical procedures, including debridement and bone grafting, are associated with concerns such as extensive trauma, restricted donor availability, and a high likelihood of recurrent infection [5]. Consequently, there is an urgent need for the development of novel, safe, and effective treatment modalities. Hydrogels, which are high-molecular polymers featuring a three-dimensional network structure, possess the ability to absorb substantial amounts of water while maintaining a definite shape. Their distinctive structure confers upon hydrogels numerous outstanding properties, including favorable biocompatibility, high water content, biodegradability, and controllable drug-release capabilities [6]. In recent years, hydrogels have been extensively investigated and applied as drug carriers in the biomedical domain [7]. TCM, a valuable asset of the Chinese nation, boasts a long-standing history and abundant experience in the treatment of bone infections [8]. Numerous TCM ingredients manifest a diverse range of effects, such as antibacterial, anti-inflammatory, and bone-repair-promoting effects, along with relatively fewer side effects [9]. Active ingredients in Traditional Chinese Medicine, such as icariin and resveratrol, can promote cell proliferation, osteogenesis, and mineralization. They can upregulate osteogenic factors like *RUNX2* and *ALP*, facilitating new bone formation. Additionally, they can induce chondrogenesis and repair bone defects [10]. The integration of TCM and hydrogels to fabricate a TCM-loaded hydrogel drug-delivery system not only maximally exploits the distinct advantages of TCM but also capitalizes on the properties of hydrogels to realize targeted drug delivery and controlled release. As a result, this integration enhances drug efficacy and minimizes the incidence of adverse reactions [11]. Consequently, the utilization of TCM-loaded hydrogels for the treatment of bone infections has emerged as one of the focal research areas in the contemporary field of bone infection management.

## 2. Classification and Advantages of Hydrogels

### 2.1. Classification of Hydrogels

**Figure 1:**
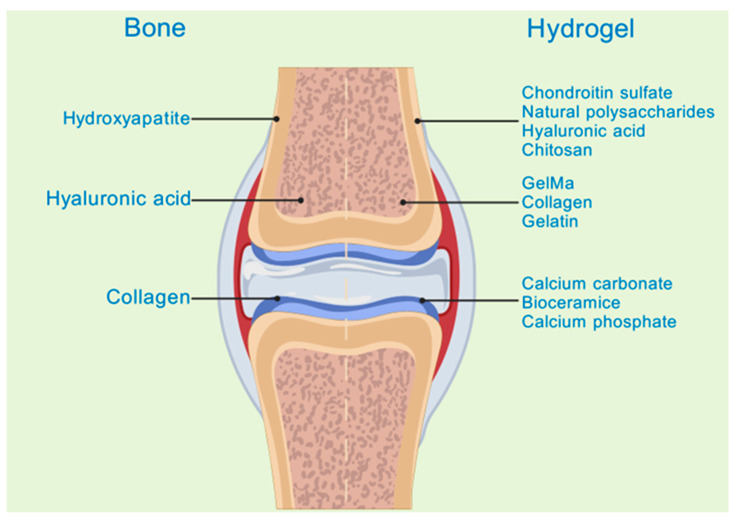
Correspondence between hydrogels and bone components. Created with BioGDP.com (accessed on 29 March 2025). Reprinted with permission from Ref. [25]. 2025, Jiang, S.

Hydrogels can be classified into three main categories based on their composition: natural hydrogels, synthetic hydrogels, and semi-synthetic hydrogels. Natural hydrogels are fabricated from natural substances. Common raw materials encompass chitosan, hyaluronic acid, sodium alginate, silk fibroin, and chondroitin sulfate, among others [12]. These natural substances exhibit outstanding biocompatibility, biodegradability, and a structure akin to that of the extracellular matrix. This similarity is advantageous for cell adhesion, proliferation, and differentiation [13] (Figure 1). For instance, sodium alginate, which is derived from marine algae, exhibits hydrophilicity, biodegradability, and biocompatibility. Sodium alginate hydrogels can be fabricated through chelation with divalent ions and find extensive applications in tissue engineering and drug delivery domains [11]. Synthetic hydrogels are predominantly fabricated from synthetic polymers, including polyethylene glycol (PEG), polyvinyl alcohol (PVA), and poly(lactic-co-glycolic acid) (PLGA), among others [14]. Synthetic hydrogels exhibit favorable mechanical properties and stability. Their characteristics can be tailored by modulating the composition and structure of the polymers [15]. For instance, polyethylene glycol (PEG) demonstrates excellent water solubility, biocompatibility, and modifiability. It is capable of forming hydrogel systems via physical, ionic, chemical, or covalent interactions and finds extensive application in tissue engineering [16]. Semi-synthetic hydrogels integrate the merits of natural and synthetic hydrogels. They are fabricated either by chemically modifying natural substances or by compounding these substances with synthetic polymers [17]. For instance, gelatin methacryloyl (GelMA), a semi-synthetic hydrogel material derived from gelatin, not only retains the biocompatibility and bioactivity inherent to gelatin but also is capable of forming a hydrogel with a certain degree of mechanical strength via photo-cross-linking. This property enables its application in constructing complex three-dimensional structures to facilitate tissue regeneration [18]. It is essential to introduce an emerging material for semi-synthetic hydrogels here: the bioactive glass hydrogel. It consists of natural gellan gum and bioactive glass particles prepared by the sol-gel method. This combination endows the hydrogel with the characteristics of both natural and synthetic materials. The bioactive glass hydrogel not only exhibits excellent biocompatibility but also effectively enhances the mechanical properties, mineralization ability, and bioactivity of the hydrogel. Research has shown that co-culturing it with rat mesenchymal stem cells can significantly promote cell attachment and mineralization. Meanwhile, synchrotron X-ray imaging analysis reveals that, although aggregation of bioactive glass particles occurs in the gellan gum hydrogel, this composite structure still demonstrates unique performance advantage [19]. It holds great promise in fields such as bone tissue engineering, offering more possibilities for the application of hydrogels in the treatment of bone infections.

The methods of shape control during cross-linking vary among different types of hydrogels. Natural hydrogels, like chitosan, rely on ionic cross-linking. Their shape can be controlled by adjusting the concentration of the cross-linking agent, reaction time, and temperature. By using molds, they can be molded as needed, which is conducive to cell-related applications [20]. Synthetic hydrogels, such as PEG hydrogels, employ photo-initiated cross-linking. They achieve precise shape regulation by controlling the illumination area and intensity and can fabricate complex microstructures for applications in fields such as microfluidics [21]. Semi-synthetic hydrogels, such as GelMA, combine the advantages of photo-cross-linking for shaping and biocompatibility. They can offer a growth environment for cells and are of great significance in tissue engineering [22]. In addition, the addition of special materials such as nanoclays and carbon nanotubes can enhance the mechanical properties and stability of hydrogels and endow them with special functions [23,24].

### 2.2. Advantages of Treating Bone Infections with Hydrogels Loaded with TCM

#### 2.2.1. Improving Drug Stability and Bioavailability

Numerous bioactive components in TCM are plagued by issues such as poor water solubility, low stability, and vulnerability to enzymatic degradation, which culminate in low bioavailability [26]. As a carrier, hydrogel can entrap the bioactive components of TCM within its three-dimensional network structure, establishing a relatively stable micro-environment. This diminishes the contact between the drug and the external milieu, thereby enhancing the drug’s stability [27]. Simultaneously, the high water content and favorable biocompatibility of hydrogels enhance the dissolution and diffusion of drugs. This, in turn, promotes drug absorption and improves bioavailability [17]. For instance, when encapsulated within a hydrogel, curcumin can evade rapid degradation and inactivation and undergo slow release. Consequently, this leads to an increase in its effective concentration and the duration of its action within the body [28]. Besides the aforementioned TCM components, there exist other TCM ingredients encapsulated within hydrogels that exhibit anti-infection, anti-inflammation, and bone-formation-promoting effects.

#### 2.2.2. Achieving Targeted Drug Delivery and Controlled Release

Hydrogels are capable of accomplishing targeted drug delivery via modification or conjugation with other materials [11]. Through the introduction of specific targeting moieties on the hydrogel surface, the hydrogel can selectively bind to the cells or tissues at the infection site. This binding event elevates the drug concentration at the infection site while minimizing damage to normal tissues [29]. Furthermore, the structure and composition of hydrogels can be precisely tailored to enable controlled drug release [30]. In accordance with the treatment requisites for bone infections, hydrogels with diverse degradation rates and drug-release profiles can be engineered. This design enables the sustained release of drugs at the infection site, maintaining an effective therapeutic concentration and enhancing the treatment efficacy. For instance, through modulating the degree of cross-linking and pore structure of the hydrogel, the drug-release rate can be regulated to achieve long-term and stable drug delivery [31]. Regulating the cross-linking and pore structure of hydrogels can modulate the drug-release rate [32]. The ideal drug-release time of the hydrogel’s entangled network is influenced by the type of bone infection, the characteristics of the drug, and the properties of the hydrogel. In the treatment of bone infections, the type of infection determines the drug-release rhythm. Acute bone infections have a rapid onset and, within 1–3 days, the hydrogel needs to rapidly release a high concentration of antibiotics. For example, a porous hydrogel loaded with vancomycin releases 60% of the drug within 24 h after implantation, which can control the deterioration of the infection. In the subsequent 2–4 weeks, the hydrogel releases the drug at a low and continuous rate, facilitating bone repair [33]. In chronic bone infections, bacteria tend to form biofilms, and bone repair is slow. Therefore, the drug release from hydrogels needs to last for months. Motasadizadeh et al. developed a pH-sensitive silk fibroin/sodium alginate hydrogel scaffold containing teicoplanin (TEC) and phenamil (PM)-loaded silk fibroin nanoparticles (PMSFNPs) for the treatment of chronic osteomyelitis. TEC and PM exhibit continuous and pH-sensitive release behavior from the hydrogel. Compared with other groups, when the bones of rats infected with methicillin-resistant *Staphylococcus aureus* are treated with the hydrogel scaffold containing PMSFNPs and TEC, the degree of infection is lower and the regeneration effect is better [34]. In the field of enhancing targeted delivery and controlled release, multifunctional hydrogels containing dopamine, magnetic, and piezoelectric nanoparticles exhibit unique advantages, bringing new opportunities for the treatment of bone infections. Thanks to its excellent adhesion, dopamine enables the hydrogel to adhere tightly to the site of bone infection, significantly increasing the local enrichment of drugs. Liu et al. prepared a dopamine-modified chitosan hydrogel, and in vitro experiments showed that dopamine modification significantly improved cell adhesion. Applying this property to the treatment of bone infections can enable the hydrogel to be more stably located in the infected area, reduce drug loss, and achieve precise targeted drug delivery [35]. Magnetic nanoparticles endow hydrogels with magnetic-responsive properties. By applying an external magnetic field, the drug-loaded hydrogels can be precisely guided to the bone infection area, achieving targeted delivery. Wu et al. introduced magnetic nanoparticles into the hydrogel system and successfully achieved drug delivery to specific sites under the action of a magnetic field. Piezoelectric nanoparticles, on the other hand, confer pressure-responsive controlled-release functions to hydrogels. When the human body moves and the bones are stressed, piezoelectric nanoparticles generate electrical signals, which change the internal microenvironment of the hydrogel and regulate the drug-release rate [36].

#### 2.2.3. Synergistically Enhancing the Therapeutic Effect

TCM usually contain multiple active ingredients, which may have synergistic effects among them, jointly exerting antibacterial, anti-inflammatory, and bone-repair-promoting effects [37]. TCM-loaded hydrogels are capable of co-encapsulating multiple bioactive components of TCM and co-releasing them at the infection site. This enables the full exploitation of the synergistic effects among these components, thereby augmenting the therapeutic efficacy [38]. For instance, the antibacterial and anti-inflammatory properties of curcumin, in combination with the bone-repair-promoting effects of other TCM components, can more efficaciously treat bone infections and facilitate the healing of bone tissue [39]. Meanwhile, the inherent properties of hydrogels, such as good biocompatibility and biodegradability, also play a role in enhancing the therapeutic effect of Traditional Chinese Medicine (TCM) and reducing the incidence of adverse reactions [40]. During the treatment of bone infections, hydrogels not only act as drug carriers but also need to support the growth and activity of osteoblasts to promote bone repair. When it comes to hydrogels serving as carriers for osteoblasts, their mechanical properties represented by G′ (storage modulus) and G″ (loss modulus) play a crucial role. In the aspect of hydrogels as osteoblast carriers, the properties represented by G′ (storage modulus) and G″ (loss modulus) are key [41]. Although there are no standard values for G′ and G″ (in Pascals) of hydrogels used as osteoblast carriers, existing research has provided us with some references. For hydrogels, to effectively support the growth and functions of osteoblasts, an appropriate balance between G′ and G″ is required [42]. The study by Li et al. indicated that cells are extremely sensitive to the viscoelasticity of their surrounding environment, and the mechanical properties of hydrogels significantly affect cell behavior. For osteoblasts, they are differentiated from stem cells, and mesenchymal stem cells differentiate in different directions on hydrogels with different stiffness. Stiffer gels are more conducive to promoting osteogenic differentiation. This means that the hydrogel used as an osteoblast carrier needs to have a relatively high stiffness to simulate the mechanical microenvironment in the body that is favorable for osteogenesis, and its G′ may be in the range of several tens to several hundreds of kPa. In terms of the loss modulus (G″), it reflects the viscosity of the hydrogel, and an appropriate G″ value plays an important role in the interaction and signal transduction between cells and the hydrogel. It can assist cells in spreading, proliferating, and differentiating on the hydrogel, and the specific value is speculated to be between several tens and several hundreds of Pascals. This is because, when G″ is within this range, the viscosity of the hydrogel is conducive to the close contact between cells and the hydrogel, ensuring signal transmission and promoting the normal physiological activities of cells [43].

### 2.3. Preparation Methods of Hydrogels Loaded with TCM

**Figure 2:**
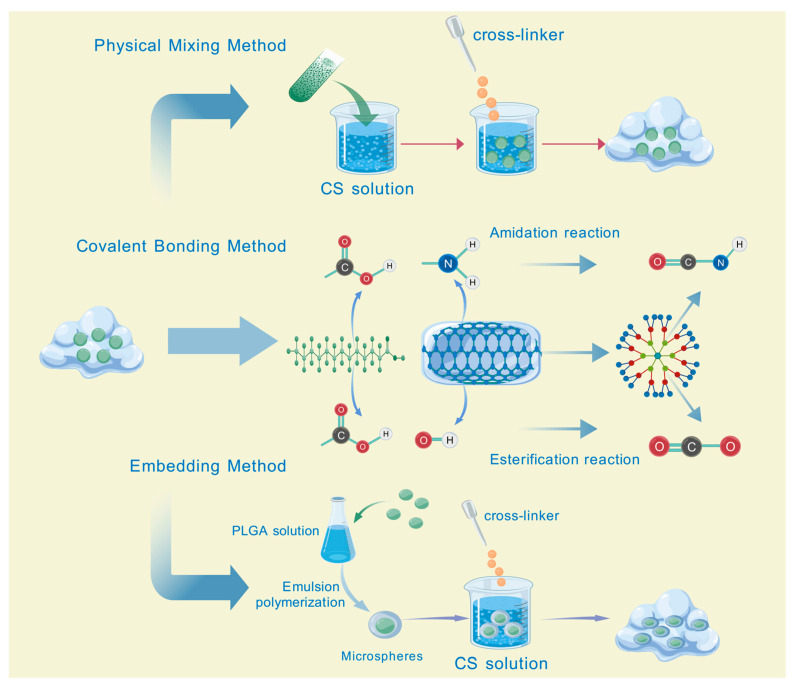
Preparation methods of hydrogels. Created with BioGDP.com (accessed on 29 March 2025). Reprinted with permission from Ref. [25]. 2025, Jiang, S.

The preparation methods of TCM-loaded hydrogels are predominantly tailored from the conventional preparation methods of hydrogels to ensure the efficient encapsulation of TCM (Figure 2). Physical mixing approach: the extracts or bioactive components of TCM are directly incorporated into the hydrogel precursor solution. Subsequently, the hydrogel is fabricated via physical cross-linking or chemical cross-linking [44]. This approach is operationally straightforward and exerts minimal influence on the bioactive components of TCM. Nevertheless, an issue of nonuniform drug distribution may arise. For instance, curcumin is directly introduced into the sodium alginate solution, following which the sodium alginate–curcumin hydrogel is synthesized by reacting with the calcium chloride solution [45]. In the physical mixing method, the pH value and chemical environment of the hydrogel precursor solution have a significant impact on the stability and dispersibility of the active ingredients of Traditional Chinese Medicine (TCM). For example, if the precursor solution is strongly acidic or strongly alkaline, it may cause the degradation or structural changes of some TCM ingredients that are sensitive to acids and alkalis, thereby reducing their biological activity. At the same time, chemical environmental factors such as the ionic strength and polarity of the solution will affect the interaction between the TCM ingredients and the hydrogel polymers. A high ionic strength may disrupt the weak interaction forces between the TCM ingredients and the polymers, resulting in uneven drug distribution and affecting the drug-loading performance and the final therapeutic effect of the hydrogel [46]. Covalent bonding approach: the bioactive components of TCM are covalently bonded to the polymer molecules of the hydrogel via chemical reactions, thereby enabling the stable loading of the drug within the hydrogel [47]. This approach enables the controlled release of the drug; however, the preparation process is relatively intricate and may impact the bioactivity of TCM. In the covalent bonding method, the reaction system’s pH value and chemical environment are crucial. They directly influence the process and outcome of the chemical reaction. A change in the pH value can impact the activity of reactive groups on both the active ingredients of Traditional Chinese Medicine and the hydrogel polymer molecules. Let’s take a reaction system with carboxyl and amino groups as an example. In an acidic environment, amino groups have a high degree of protonation. This high protonation makes it difficult for amino groups to undergo a condensation reaction with carboxyl groups to form covalent bonds. Conversely, in an alkaline environment, carboxyl groups tend to exist as carboxylates. This form reduces the reaction activity between carboxyl and amino groups, also affecting the formation of covalent bonds [48]. Embedding technique: TCM is encapsulated within the hydrogel to form a microcapsule-like structure [49]. This approach can shield TCM from the external milieu and enhance the drug’s stability. For instance, the emulsion polymerization technique is employed to encapsulate TCM components within poly(lactic-co-glycolic acid) (PLGA) microspheres. Subsequently, these microspheres are dispersed in the hydrogel matrix to fabricate an embedded hydrogel drug-delivery system [27]. In the embedding technique, such as in the process of preparing drug-loaded microspheres by emulsion polymerization and embedding them in hydrogels, the actual pH value and chemical environment play a crucial role in the formation and stability of the microspheres [50]. In the emulsion polymerization reaction, the pH value can affect the performance of the emulsifier and the polymerization reaction of the monomers. Acidic or alkaline conditions may change the hydrophilic–lipophilic balance of the emulsifier, leading to a decrease in the stability of the emulsion, which, in turn, affects the particle size distribution and encapsulation efficiency of the microspheres. Impurities in the chemical environment, such as metal ions, may trigger side reactions, interfere with the normal polymerization process of the microspheres, and affect the encapsulation effect of the active ingredients of Traditional Chinese Medicine. Moreover, when dispersing the drug-loaded microspheres into the hydrogel matrix, the chemical environment of the hydrogel matrix, such as the concentration of the cross-linking agent and the concentration of the polymer, will affect the interaction between the microspheres and the hydrogel. If the interaction is too weak, the microspheres may aggregate or leak in the hydrogel, reducing the performance of the hydrogel and the controllability of drug release.

### 2.4. The Release Mechanisms of Traditional Chinese Medicine from Hydrogels

The release of Traditional Chinese Medicine from the hydrogel matrix is a crucial link for exerting its therapeutic effects, which is mainly achieved through diffusion, swelling, and degradation. Hydrogels possess a porous three-dimensional network structure. The active ingredients of Traditional Chinese Medicine can diffuse from the interior of the hydrogel to the exterior by virtue of the concentration gradient [51]. For example, some small-molecule components of Traditional Chinese Medicine can relatively easily diffuse into the surrounding environment through the pores of the hydrogel. The diffusion rate is influenced by factors such as the pore size of the hydrogel, its porosity, and the size of the drug molecules. When the hydrogel has a relatively large pore size and the drug molecules are small, the diffusion speed is relatively fast; conversely, it is slower [52]. Hydrogels will swell after absorbing water, and their network structure will become more porous. This increases the diffusion path of the components of Traditional Chinese Medicine, which is beneficial for drug release [53]. The degradation mechanisms of hydrogels are diverse. Photodegradation relies on light of specific wavelengths and doses to make photo-labile groups react. Enzymatic degradation achieves site-specific degradation by leveraging the concentration differences of enzymes in different cells or tissues. Hydrolytic degradation occurs when water is present and the polymer contains easily hydrolyzable groups. It is affected by multiple factors and may exhibit autocatalysis. Redox-induced degradation depends on the cleavage of disulfide bonds in a reducing environment. Mechanical degradation breaks thermally stable cyclic bonds through special mechanical forces. Degradation based on dynamic covalent bonds makes covalent bonds change reversibly under external stimuli. These different degradation mechanisms endow hydrogels with unique applications in numerous fields [54]. Wang et al. developed a pH-responsive hydrogel. This pH-responsive degradation property enables the hydrogel to accelerate degradation in the acidic microenvironment of tumors and precisely release drugs.

### 2.5. The Stimuli for the Gel-Sol Transition of Hydrogels

There are various stimuli for the gel-sol transition of hydrogels to achieve the release of Traditional Chinese Medicine, mainly including temperature stimuli, pH stimuli, enzyme stimuli, and magnetic field stimuli. Temperature-sensitive hydrogels can undergo a gel-sol transition under specific temperature changes. Poly(N-isopropylacrylamide) hydrogel is a typical temperature-sensitive hydrogel. Yang et al. used N-isopropylacrylamide as a temperature-sensitive monomer and synthesized a series of temperature-sensitive hydrogels through free-radical polymerization. At different temperatures, their internal structures and properties will change. This temperature sensitivity endows them with potential application values in fields such as drug sustained release, material separation, and enzyme immobilization [55]. The pH values vary in different parts of the body. pH-sensitive hydrogels designed by leveraging this characteristic can achieve targeted drug release. Peng et al. proposed a new method for the release of lipophilic drugs based on a dual-pH-responsive hydrogel actuator. This method uses a dual-pH-controlled capsule switch to encapsulate and release drugs. Inspired by the deformation mechanism of the leaves of the sundew plant, the capsule switch has a double-layer structure, which is composed of two pH-responsive hydrogels covalently connected by a silane coupling agent. It can bend synergistically in acidic or alkaline environments to achieve the “opening” action of the capsule switch. Adding parallel elastomer stripes on one side of the hydrogel bilayer enables the actuator to perform various movements such as bending, twisting, and rolling. In vitro lipophilic drug release tests verified the feasibility of this method, providing new ideas for the development of multiple drug delivery systems. Enzyme-responsive hydrogels are a special type of stimuli-responsive hydrogels that can respond to specific enzymes and change their properties. Through the interaction between enzymes and chemical groups in the hydrogel, reversible or irreversible changes in the chemical and physical properties of the hydrogel are achieved, thereby controlling drug release. For example, matrix metalloproteinases (MMPs) can enzymatically cleave specific peptide-segment cross-linkers in the hydrogel, causing the polymer to degrade and release anticancer drugs. Glucose oxidase (GO) can convert glucose into gluconic acid, triggering a pH change that indirectly affects the properties of the hydrogel to control insulin release [56]. For magnetic hydrogels containing magnetic nanoparticles, under the action of an external magnetic field, the magnetic nanoparticles will interact with each other, causing changes in the microstructure of the hydrogel, which, in turn, affects the release of drugs. Zhao et al. synthesized a magnetic field-responsive hemicellulose hydrogel. This hydrogel exhibits excellent adsorption and controlled-release properties. Under the action of an external magnetic field, the magnetic nanoparticles inside it will respond, prompting changes in the hydrogel structure, thus achieving the regulation of drug release [57].

## 3. Mechanisms of TCM in the Treatment of Bone Infections

**Figure 3:**
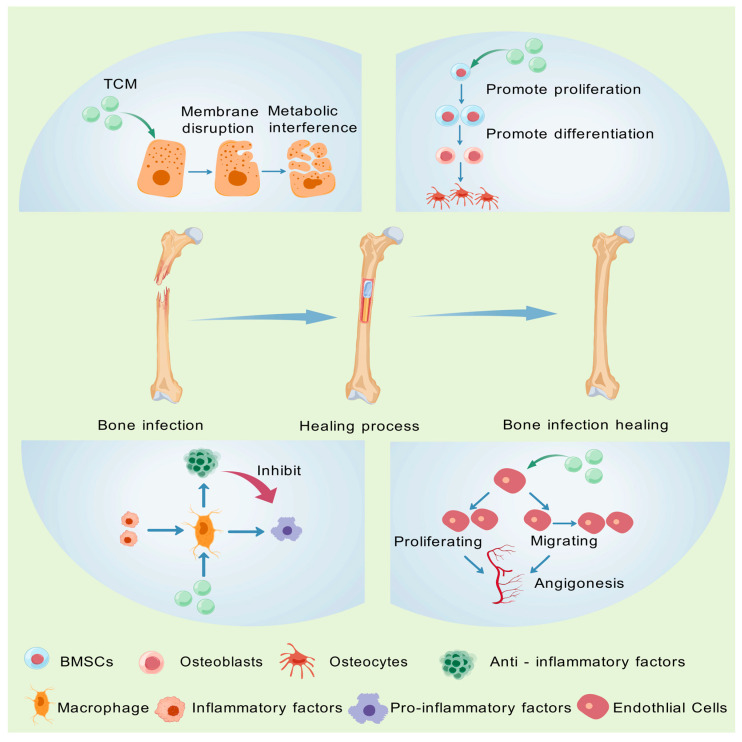
Mechanism of action of CHM components. Created with BioGDP.com (accessed on 29 March 2025). Reprinted with permission from Ref. [25]. 2025, Jiang, S.

In the realm of bone infection treatment, the components of TCM demonstrate distinctive and diverse therapeutic merits. They primarily exert their effects via four pivotal mechanisms: antibacterial action, anti-inflammatory activity, promotion of osteoblast proliferation, and angiogenesis (Figure 3).

### 3.1. Antibacterial Activity

In the context of bone disease treatment, infectious complications severely hinder bone repair [5]. Components such as Flos *Lonicerae Japonicae* [58] and *Cortex Phellodendri* [59] in TCM possess antibacterial activities. Chlorogenic acid and flavonoids present in Flos *Lonicerae Japonicae* are capable of disrupting the integrity of bacterial cell membranes. They further interfere with bacterial energy metabolism and protein synthesis processes. As a result, these compounds exhibit substantial inhibitory effects against *Staphylococcus aureus*, Escherichia coli, and other relevant bacteria [60]. Berberine contained in *Cortex Phellodendri* has the ability to inhibit the *tarO* gene, thereby affecting the cell-wall synthesis of methicillin-resistant *Staphylococcus aureus* [61]. Soltani et al. fabricated a chitosan/alginate scaffold and incorporated berberine along with Berberis fruit extract. The research findings indicated that berberine could enhance the osteogenic differentiation capacity of mesenchymal stem cells within the chitosan/alginate scaffold. It potentially promoted the synthesis and mineralization of the bone matrix by upregulating the expression of genes such as *ALP*, *COL1A2*, and *Runx2*. Consequently, berberine played a crucial role in bone repair [62]. Chen et al. synthesized and characterized a gelatin–chitosan-based film impregnated with berberine hydrochloride and polypyrrole. This film exhibited potent antibacterial activity against Gram-positive bacteria, such as *Staphylococcus aureus*. The interaction between the positive charge of polypyrrole and the negative charge on the bacterial surface augmented the antibacterial effect. Additionally, the film demonstrated favorable antioxidant activity and could efficiently scavenge DPPH free radicals, thereby reducing the adverse impact of oxidative stress on wound healing [63]. Zhou et al. synthesized cyclodextrin-grafted chitosan (CD-g-CS) and fabricated the BBH@CD-g-CS/GP-SHC hydrogel. This hydrogel was shown to be degradable under simulated in vivo conditions and demonstrated significant antibacterial activity against *Escherichia coli* and *Staphylococcus aureus*. The antibacterial effect persisted for 48 h against *Escherichia coli* and 4 days against *Staphylococcus aureus*. Moreover, the maximum inhibitory diameter against *Staphylococcus aureus* could reach up to 28 mm [64]. When these TCM are incorporated into hydrogels, the hydrogels function as drug depots, facilitating the slow and continuous release of drugs. This enables the antibacterial components to maintain a high local concentration and exert antibacterial effects over an extended period. Moreover, research has demonstrated that berberine can inhibit bacterial growth and replication by suppressing bacterial-division-related proteins [65]. The incorporation of antibacterial components into hydrogels facilitates their gradual release at the infection locus. This sustained release phenomenon inhibits bacterial growth over a prolonged time frame, thereby creating a conducive antibacterial milieu for the treatment of bone diseases.

### 3.2. Promotion of Osteoblast Proliferation

The promotion of osteoblast proliferation holds substantial significance in the treatment of bone diseases. Cyaonoside A, an active component in the TCM Achyranthes bidentata, is capable of binding to the receptors on the surface of osteoblasts. This binding event activates the mitogen-activated protein kinase signaling pathway, thereby promoting the proliferation and differentiation of osteoblasts and upregulating the expression of key cartilage-specific genes such as ACAN, COL2A, and *SOX9* [66]. Icariin, an active component in Epimedium, is capable of inducing the proliferation, differentiation, and mineralization of osteoblasts. This occurs via the activation of extracellular-signal-regulated kinases and c-Jun N-terminal kinases (JNK) signals, which are mediated by estrogen receptors [67]. Modern investigations have demonstrated that icariin modulates the equilibrium of bone metabolism via multiple targets and pathways. It has been extensively explored in the domains of osteoporosis, osteoarthritis, and fracture healing [68]. Liu et al. engineered a high-strength double-network silk fibroin hydrogel impregnated with icariin and seeded with bone marrow mesenchymal stem cells. This hydrogel exhibited high mechanical strength and favorable biocompatibility. It was capable of promoting the proliferation and differentiation of BMSCs, inhibiting osteoclastogenesis, and facilitating the regeneration of rat femoral injuries [69]. Li et al. fabricated a nanoparticle/hydrogel hybrid system capable of continuously delivering IL-10 and icariin for bone defect repair. This hybrid system exhibited mechanical strength, injectability, and photo-cross-linkability. The rapid release of IL-10 exerted immunomodulatory activity, whereas the sustained release of icariin promoted the osteogenic differentiation of bone-marrow-derived mesenchymal stem cells. The system demonstrated a synergistic effect on bone remodeling in a severe rat cranial defect model [70]. Zeng et al. engineered a “mussel-mimicking” multi-responsive hydrogel-mediated co-delivery system for exosomes (Exos) and icariin (ICA). Exosomes derived from bone marrow mesenchymal stem cells were utilized as carriers for icariin to enhance its cellular delivery efficiency. Subsequently, the “mussel-mimicking” multi-responsive hydrogel served as a delivery vehicle for the drug-loaded exosomes. This setup formed a drug depot within the joint cavity and extended the residence time of exosomes in the body. The system exerted a synergistic effect in promoting cell proliferation and migration while inhibiting the secretion of matrix metalloproteinases, thereby accelerating cartilage repair [71]. Crocin, an active constituent in Crocus sativus, is capable of promoting the proliferation of MC3T3-E1 cells. In addition, following osteogenic induction and differentiation, crocin increases the mRNA expression of *RUNX2*, type I collagen, alkaline phosphatase, and osteocalcin in cells in a dose-dependent fashion [72]. When delivered via hydrogels, these TCM components can act on osteoblasts more efficaciously, thereby promoting the repair and regeneration of bone tissue. In addition to the functions of promoting the proliferation, differentiation, and mineralization of osteoblasts mentioned above, Traditional Chinese Medicine also plays an important role in maintaining the calcium–phosphorus ratio. In bone tissue, the stability of the calcium–phosphorus ratio is crucial for the normal structure and function of bones. Numerous studies have shown that the active ingredients of Traditional Chinese Medicine can affect calcium–phosphorus metabolism through multiple pathways, thereby maintaining the calcium–phosphorus ratio. For example, Gao et al. pointed out that Traditional Chinese Medicine components such as icariin can enhance the ability of osteoblasts to uptake and utilize calcium and phosphorus while promoting the proliferation of osteoblasts. During the process of bone mineralization, osteoblasts deposit the absorbed calcium and phosphorus into the bone matrix to form hydroxyapatite crystals. This not only contributes to the mineralization of the bone matrix but also maintains the stability of the calcium–phosphorus ratio in bone tissue. When bone tissue is damaged or the calcium–phosphorus ratio is unbalanced, icariin promotes an increase in osteoblast activity, prompting more calcium and phosphorus to be deposited in the bone tissue, thus restoring the normal level of the calcium–phosphorus ratio [10]. Duan et al. pointed out that *Drynaria fortunei* can increase the body’s absorption of calcium, promote the transportation of calcium to bone tissue, enhance bone mineralization, maintain the balance of bone remodeling, and thus regulate calcium–phosphorus metabolism [73].

### 3.3. Anti-Inflammation

Inflammation persists throughout the onset and progression of bone diseases. Excessive inflammation can inflict damage on bone tissue [74]. Components in TCM, such as flavonoids and alkaloids, display notable anti-inflammatory activities [75]. Baicalin, an active component in *Scutellaria baicalensis*, is capable of inhibiting the levels and expression of inflammatory factors including TNF-α, IL-1β, and IL-6 [76]. Triptolide, an active constituent in Tripterygium wilfordii, has the ability to modulate the functions of immune cells. It can inhibit the activation and proliferation of T lymphocytes and diminish the infiltration of inflammatory cells [77]. Curcumin, an active ingredient in Curcuma longa, is able to mitigate the inflammatory response through the inhibition of the activities of inflammation-related enzymes, including cyclooxygenase-2 and inducible nitric oxide synthase [78]. Once incorporated into hydrogels, these TCM components can be continuously released at the inflammatory site, effectively dampening the inflammatory response and facilitating the treatment of bone diseases. Kalantarnia et al. fabricated curcumin-loaded polycaprolactone (PCL) microspheres and combined them with chitosan, β-glycerophosphate, and bacterial cellulose nanofibers to construct a hydrogel. This hydrogel exhibited an appropriate gelation time, porosity, and pore size, which were conducive to cell growth. The hydrogel containing drug-loaded microspheres enabled the slow release of curcumin. It demonstrated antibacterial activity against *Staphylococcus aureus* and *Escherichia coli* and manifested good cartilage regeneration effects in in vivo experiments [79]. Wu et al. fabricated the GMDE/UIO-66@Cur hydrogel. This hydrogel exhibited favorable mechanical properties and swelling ratio and was capable of effectively absorbing wound exudate. Curcumin could be continuously released from the hydrogel over a period of 96 h, with an accumulated release rate reaching up to 66.35%. It demonstrated a significant antibacterial effect against *Escherichia coli* and *Staphylococcus aureus*. Cell-based experiments indicated that this hydrogel possessed good biocompatibility and could promote cell migration and angiogenesis. In in vivo experiments, following 14 days of treatment with the GMDE/UIO-66@Cur hydrogel, the wound-healing rate was significantly higher than that of the control group. The hydrogel could effectively mitigate the inflammatory response and facilitate the growth of granulation tissue, angiogenesis, and collagen deposition [80]. Research has demonstrated that quercetin promotes the proliferation and survival of bone marrow mesenchymal stem cells (BMSCs) and augments their osteogenic differentiation capacity by activating the PI3K/Akt signaling pathway. Conversely, quercetin inhibits the activation of the NF-κB signaling pathway, diminishes the production of inflammatory factors, and thereby alleviates the inflammatory response, establishing a favorable microenvironment for bone regeneration [81]. Ding et al. fabricated a hydrogel via the Schiff-base reaction between CMCS and Odex and impregnated it with quercetin. Post-quercetin impregnation, the antioxidant and antibacterial properties of the hydrogel were augmented. Additionally, the hydrogel exhibited self-healing and injectability characteristics, along with good cell compatibility, demonstrating substantial potential in wound-healing treatment [82]. Wang et al. fabricated an antibacterial hydrogel incorporating quercetin, and its performance was markedly enhanced. The robust binding force between quercetin and the hydrogel matrix augmented the degree of cross-linking within the hydrogel, thereby leading to a substantial improvement in its antibacterial properties. This hydrogel demonstrated a potent inhibitory effect against *Escherichia coli* and *Staphylococcus aureus* and could effectively disrupt the morphological structure of bacteria [83].

### 3.4. Angiogenesis Promotion

Angiogenesis is essential for bone repair and regeneration [84]. Tanshinones, the active components in *Salvia miltiorrhiza*, promote angiogenesis via regulating the miR-499-5p/PTEN signaling pathway [85]. Several investigations have revealed that tanshinone IIA, a lipophilic phenanthraquinone compound extracted from the Chinese herb *Salvia miltiorrhiza*, can inhibit osteoclastogenesis and bone loss in ovariectomized mice. The underlying mechanism is linked to the inactivation of the NF-κB and Akt signaling pathways. Tanshinone IIA exhibits multiple biological activities, including antibacterial, anti-inflammatory, angiogenesis-promoting, and bone-repair capabilities [86]. Chen et al. successfully fabricated a silk fibroin scaffold loaded with tanshinone IIA. This scaffold is capable of enhancing chondrocyte activity, alleviating oxidative stress, and effectively repairing cartilage defects [87]. Li et al. engineered a hydrogel-hybrid scaffold composed of polycaprolactone (PCL)–meniscus extracellular matrix (MECM) loaded with sodium tanshinone IIA sulfonate (STS) to facilitate meniscus regeneration and cartilage protection. By creating a microenvironment that is favorable for cell proliferation and differentiation, this scaffold promotes M2 polarization and shields meniscus-derived fibrochondrocytes (MFC) from inflammatory stimuli, thereby enhancing meniscus regeneration and cartilage protection. Additionally, a chitosan nanogel loaded with tanshinone IIA was investigated. This hydrogel effectively inhibits biofilm formation and disrupts the biofilm structure [88]. Tetramethylpyrazine, an active ingredient in Ligusticum chuanxiong, is capable of enhancing hemorheology. It reduces blood viscosity, increases local blood flow, and supplies sufficient nutrients to bone tissue, thereby promoting angiogenesis [89]. Safflower yellow pigment, an active constituent in Carthamus tinctorius, facilitates angiogenesis in bone marrow endothelial cells through the glycolysis-mediated VEGFA/VEGFR2 signaling pathway [90]. Hydrogels enable the slow release of these TCM components into the surrounding tissues. This release regulates the expression of cytokines and growth factors, recruits angiogenesis-related cells, and collectively promotes angiogenesis, thereby accelerating the repair and regeneration of bone tissue.

### 3.5. Promotion of Nerve Repair

During the bone repair process, neurogenesis plays a crucial role. Nerves can not only provide nutritional support for bone tissue but also promote the proliferation and differentiation of bone cells, as well as the synthesis and mineralization of the bone matrix by regulating cell activities and signaling pathways [91,92]. In recent years, studies have found that multiple components of Traditional Chinese Medicine have shown significant efficacy in promoting neurogenesis, providing new directions for the treatment strategies of bone repair. Existing research indicates that the active ingredients in Traditional Chinese Medicine can promote neurogenesis through multiple mechanisms, thereby contributing to bone repair. Ginsenoside is one of the main active ingredients in ginseng. Research has found that ginsenoside Rg1 can induce the transformation of reactive astrocytes in rats into neuron-like cells and promote the recovery of motor function in rats with spinal cord injuries. Its mechanism is related to the inhibition of the Notch/Stat3 signaling pathway. This result is of great significance for promoting nerve repair and, thus, bone repair. During bone repair, healthy nerves can provide nutrition for bone tissue and regulate the activity of bone cells. Ginsenoside Rg1 promoting nerve repair can improve the microenvironment of bone repair. Its regulation of the Notch/Stat3 signaling pathway may affect the behavior of bone-related cells. Enhancing the antiapoptotic ability of cells also helps to maintain the activity of bone-repair-related cells, thus indirectly promoting the repair and regeneration of bone tissue [93]. Wang et al. discovered that β-elemene can enhance the expression of growth-associated protein 43 (GAP-43) by inhibiting the activation of RhoA kinase, thereby promoting neurite growth. During the bone repair process, some signaling pathways and molecular mechanisms are interconnected. The RhoA kinase signaling pathway plays an important role in processes such as cell proliferation, differentiation, and migration, and the same is true for bone cells. The regulation of this signaling pathway by β-elemene may not only affect nerve cells but also potentially impact cells involved in bone repair, such as osteoblasts and bone marrow mesenchymal stem cells, promoting the proliferation, differentiation, and migration of these cells and, thus, directly or indirectly promoting bone repair [94].

Besides the aforementioned components, a plethora of other TCM components, upon being loaded onto hydrogels, display distinct functional characteristics and reveal substantial potential application value in the realm of bone infection treatment (Table 1).

## 4. Challenges and Prospects

Despite the significant potential shown by hydrogels loaded with TCM in treating bone infections, they encounter numerous challenges. In-depth research across multiple facets is essential to propel the advancement of this field. For hydrogel performance optimization, current drug-loading capacities and release properties are sub-par. Future work should optimize formulation and preparation processes, achievable by choosing appropriate materials, improving cross-linking methods, and adjusting polymer ratios to enhance hydrogel–TCM component interactions, thus precisely controlling drug loading and release. In terms of biosafety and immunogenicity, the interaction mechanisms among hydrogels, drugs, cells, and tissues remain unclear, posing immunological risks. Advanced techniques should be used for comprehensive research and an evaluation system established to screen suitable materials and reduce immunological risks, ensuring clinical safety. Clinical translation is fraught with difficulties, as existing bone infection models differ greatly from clinical reality, making experimental results lack clinical relevance. There is a need to construct more clinically relevant models considering various factors and strengthen the connection between preclinical research and clinical trials to promote research translation. The research on TCM active ingredients needs strengthening due to TCM’s complex composition and unclear screening of active ingredients and their action mechanisms. Technologies like high-throughput screening should be used to clarify active ingredients and their synergistic mechanisms, providing a theoretical basis for optimizing the hydrogel-based drug delivery system. Interdisciplinary collaboration is crucial for addressing these challenges. Multiple disciplines, such as materials science, pharmacy, and medicine, need to co-operate closely, leveraging their respective advantages to promote the translation of this treatment method from lab to clinic and offer more effective treatment options for bone infection patients.

## 5. Conclusions

Bone infections significantly compromise patients’ health, while traditional treatment modalities exhibit certain limitations. TCM holds great promise in the treatment of bone infections. It encompasses multiple active constituents and exerts functions, including antibacterial, anti-inflammatory, and bone-repair-promoting effects. Incorporating TCM components into hydrogels enables the exploitation of hydrogel advantages for precise drug delivery and sustained release, thus augmenting the therapeutic efficacy. For instance, components such as tanshinone IIA and curcumin can effectively combat bone infections and facilitate bone healing. Nevertheless, at present, this technology confronts challenges, such as the optimization of hydrogel performance. Future research endeavors must be intensified to facilitate its clinical translation and offer more effective treatment alternatives for patients with bone infections.

## Figures and Tables

**Table 1:** Hydrogel systems loaded with TCM components.

TCM	Molecular Formula	Intermolecular Forces	Function	Ref.
Baicalin	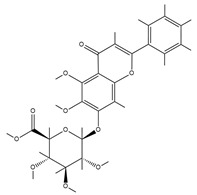	Baicalin contains active functional groups such as hydroxyl and carboxyl groups. It can combine with the corresponding groups on the polymer chains of hydrogels through covalent bonds, such as ester bonds.	Antibacterial and anti-inflammatory, promoting wound healing.	[95]
Hypericin	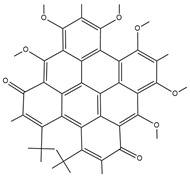	Hypericin molecules possess polar groups, such as hydroxyl groups. These groups can form hydrogen bonds with hydrophilic groups in hydrogels, such as carboxyl and hydroxyl groups.	Antibacterial, anti-inflammatory, and reduction in scar formation.	[96]
Ginsenoside	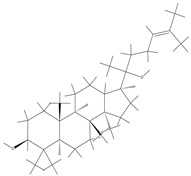	Ginsenoside molecules contain functional groups like hydroxyl and carboxyl groups, which can bind to the active groups on hydrogel polymer chains via covalent bonds.	Anti-inflammatory, antioxidant, antibacterial, and promoting angiogenesis.	[97]
Rhein	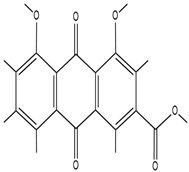	Rhein molecules have certain hydrophobicity and planar structure. The three-dimensional network structure of hydrogels has pores and hydrophobic regions, which can adsorb rhein inside through physical interactions such as van der Waals forces.	Induce the differentiation of bone marrow mesenchymal stem cells (BMSCs) into osteoblasts.	[98]
Epigallocatechin gallate	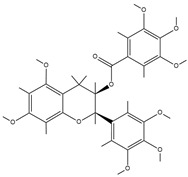	Polar groups such as hydroxyl and carboxyl groups in hydrogels can form numerous hydrogen bonds with the phenolic hydroxyl groups of EGCG. Additionally, the benzene ring structure of EGCG may also interact with the aromatic-structured parts in hydrogels through π–π stacking.	Anti-inflammation, anti-oxidation, and promotion of cartilage regeneration.	[99]
Asiatic acid	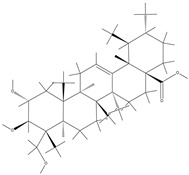	Asiatic acid molecules contain polar groups such as hydroxyl groups, which can form hydrogen bonds with hydrophilic groups in hydrogels. Meanwhile, its partial carbon-chain structure is hydrophobic and can interact hydrophobically with the hydrophobic regions of hydrogels.	Antioxidant, anti-inflammatory, and antibacterial.	[100]
Bletilla striata polysaccharide	Not single component	\	Antibacterial, antioxidant, and promote angiogenesis.	[101]
Poria cocos triterpenes extract	Not single component	\	Anti-inflammatory, promote the polarization of M2 macrophages and angiogenesis.	[102]
Puerarin	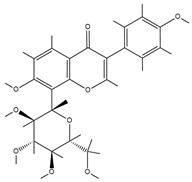	Puerarin molecules contain multiple polar groups such as hydroxyl groups. Hydrogels are also rich in hydrophilic groups like hydroxyl and amino groups. They can interact with each other through hydrogen bonds.	Antibacterial, anti-inflammatory, and promotion of cell migration and proliferation.	[103]
Naringin	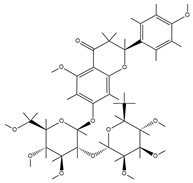	Naringin molecules contain multiple polar groups such as hydroxyl groups. There are also a large number of hydrophilic groups like hydroxyl and carboxyl groups in hydrogels. They can combine with each other through hydrogen bonds.	Antibacterial, hemostatic, promoting bone regeneration, and facilitating neurovascularization.	[104]
Oleanolic acid	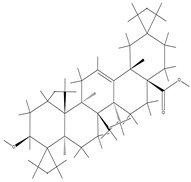	Oleanolic acid has certain hydrophobicity. If there are hydrophobic regions in hydrogels, they can bind to oleanolic acid through hydrophobic interactions.	Anti-inflammation and promotion of cartilage regeneration.	[105]
Triptolide	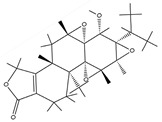	Triptolide has certain hydrophobicity. If hydrogels contain hydrophobic regions or segments, they can bind to triptolide through hydrophobic interactions.	Anti-inflammatory, promotes the proliferation and differentiation of articular chondrocytes.	[106]
Tetramethylpyrazine	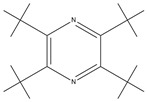	Tetramethylpyrazine itself has relatively weak polarity. However, if there are a large number of strongly polar groups such as hydroxyl and carboxyl groups in hydrogels, and the nitrogen atoms in tetramethylpyrazine molecules can act as hydrogen-bond acceptors, weak hydrogen bonds can form between them and the hydroxyl donors in hydrogels.	Anti-inflammatory, improves local blood circulation, and promotes injury repair.	[107]
